# Supplementary material for: TastepepAI: An artificial intelligence platform for taste peptide de novo design
Source: PLoS Comput Biol. 2025 Oct 16;21(10):e1013602. doi: 10.1371/journal.pcbi.1013602 (PMC12543283; doi:10.1371/journal.pcbi.1013602)
Supplement: S1 File — (ZIP) [file pcbi.1013602.s006.zip › Supplementary Data 2/TaPep13 MS.doc]

# Mass Spectrometry Report

| [M+H]+  [M+H]+  [M+2H]2+ | | | | | | | |
| --- | --- | --- | --- | --- | --- | --- | --- |
|  | Sample Description | | Instrument | SHIMADZU LCMS-2020 | | | |
|  | Analyzed date: | 2024/9/24 | Probe: | ESI | | Probe Bias: | ＋4.5kv |
|  | Analyst: | Huang | Nebulizer Gas Flow: | 1.5L/min | | Detector: | 1.2kv |
|  | Sample: | FCl-NJP93314 TaPep13 YS-6 | CDL: | -20.0v | | T. Flow: | 0.2ml/min |
|  | M.W.: | 825.86 | CDL Temp.: | 250 | ^o^C | B. Conc.: | 50%H_2_O/50%ACN |
|  | Lot. No.: | P240913-MX1172217 | Block Temp.: | 400 | ^o^C |  |  |
